# Machine Learning-Assisted SERS Platform for Rapid and Quantitative Discrimination of Shiga Toxin-Producing *E. coli* Serotypes

**DOI:** 10.3390/bios15110740

**Published:** 2025-11-04

**Authors:** Yuting Liu, Jiyu Feng, Xinyi Chen, Mingyu Cheng, Jinglan Zhang, Xu Ye, Yiping Zhao, Bin Ai

**Affiliations:** 1 Chongqing Key Laboratory of Bio-Perception & Intelligent Information Processing, School of Microelectronics and Communication Engineering, Chongqing University, Chongqing 400044, China; 2 Department of Physics and Astronomy, The University of Georgia, Athens, GA 30602, USA

**Keywords:** surface-enhanced Raman scattering (SERS), *Escherichia coli* serotypes, silver nanorod arrays, vancomycin functionalization, pathogen detection

## Abstract

Rapid, sensitive, and specific detection of pathogenic *Escherichia coli* serotypes is crucial for food safety and public health. Here, we present a surface-enhanced Raman scattering (SERS) platform utilizing highly ordered silver nanorod (AgNR) arrays functionalized with vancomycin for efficient and selective bacterial capture. The system enables multiplexed, high-throughput analysis using a portable Raman spectrometer, achieving direct molecular fingerprinting of seven clinically relevant *E. coli* serotypes. Systematic optimization of AgNR length and vancomycin coating maximized SERS enhancement and capture efficiency. Advanced data analysis with linear discriminant analysis (LDA) provided robust discrimination among all serotypes and concentrations, achieving up to 100% classification accuracy in single-concentration models and an overall accuracy of 98.41% when all concentrations and serotypes were evaluated jointly. This integrated SERS approach demonstrates significant promise for rapid, on-site bacterial diagnostics and quantitative pathogen monitoring, paving the way for practical applications in food safety and clinical microbiology.

## 1. Introduction

*Escherichia coli* (*E. coli*) comprises a genetically diverse group of Gram-negative bacteria, among which certain pathogenic strains, especially Shiga toxin-producing *E. coli* (STEC), pose serious risks to food safety and public health worldwide [1,2]. Rapid and accurate identification of these strains is critical for preventing foodborne outbreaks and guiding timely clinical interventions. Conventional detection methods such as bacterial culture, polymerase chain reaction (PCR), and immunoassays remain the gold standard in diagnostic laboratories [3,4,5,6], but they are often hampered by long turnaround times, labor-intensive workflows, and reduced sensitivity or specificity in complex food or environmental matrices. These limitations reveal the need for alternative platforms that can deliver fast, sensitive, and multiplexed bacterial detection at the point of care.

Surface-enhanced Raman scattering (SERS) has emerged as a powerful tool for bacterial diagnostics due to its ability to provide label-free molecular fingerprinting with ultrahigh sensitivity and minimal sample preparation [7,8,9]. By leveraging the plasmonic enhancement of Raman signals near nanostructured metallic surfaces—typically silver or gold—SERS enables the detection of subtle biochemical differences among microbial species and even closely related strains [10,11,12]. As a result, SERS is particularly attractive for *E. coli* serotyping, where traditional techniques often lack the resolution to discriminate between highly similar variants. The field has rapidly evolved from proof-of-concept demonstrations to practical applications, with growing emphasis on portable instrumentation and real-world sample analysis [13,14,15].

However, several technical challenges still hinder the widespread adoption of SERS in routine pathogen monitoring. Key among these are the reproducibility and robustness of SERS-active substrates, as well as their capacity to generate consistent signal enhancement across multiple measurements [16]. Many nanostructured substrates suffer from batch-to-batch variability and limited long-term stability, which compromise both analytical sensitivity and quantification reliability [17,18]. In this context, silver nanorod (AgNR) arrays fabricated via glancing angle deposition (GLAD) have attracted attention due to their tunable morphology, high density of electromagnetic “hot spots” (enhancement factor > 10^8^), and excellent fabrication reproducibility (a batch-to-batch variability < 15%) [19,20,21]. These AgNR substrates have been successfully used for bacterial detection [12,15,20,22,23], but their application to the multiplexed identification and quantification of STEC strains remains unexplored. Furthermore, as SERS generates high-dimensional and often complex spectra, effective data interpretation is essential for achieving accurate bacterial classification. While previous studies have applied simple statistical or chemometric tools, such as principal component analysis (PCA), these unsupervised methods often fall short when resolving closely related serotypes or simultaneously accounting for concentration-dependent signal variation. More advanced approaches—particularly supervised machine learning algorithms—offer the potential to enhance classification accuracy, support quantitative analysis, and enable real-time decision-making in practical settings [24,25].

In this study, we present a fully integrated SERS-based biosensing platform for the rapid and quantitative discrimination of seven clinically relevant *E. coli* serotypes, including multiple STEC strains. By optimizing the length of GLAD-fabricated Ag nanorods and systematically tuning vancomycin (VAN)-mediated surface functionalization, we achieved efficient bacterial capture and uniform signal enhancement. A portable Raman spectrometer coupled with a high-throughput sample handling module was used for rapid spectral acquisition. To robustly classify serotypes and quantify bacterial concentrations, we implemented linear discriminant analysis (LDA), which provided superior performance over traditional methods. This combined experimental and computational framework addresses key bottlenecks in current SERS-based detection strategies and offers a scalable, field-deployable solution for food safety, clinical diagnostics, and environmental pathogen surveillance.

## 2. Results and Discussion

### 2.1. Measurement Workflow

**Figure 1:**
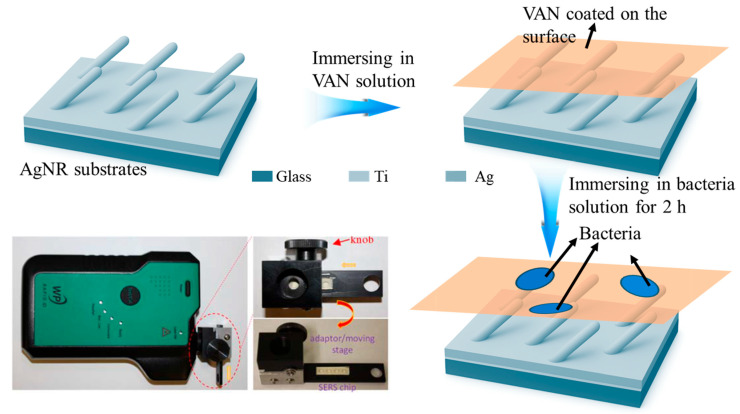
Schematic illustration of the SERS-based workflow for *E. coli* serotype detection. (Step 1) fabrication of AgNR substrates by GLAD; (Step 2) functionalization of AgNRs with VAN to enable selective bacterial capture; (Step 3) incubation of VAN-coated substrates with *E. coli* suspensions for targeted binding; (Step 4) portable Raman spectrometer integrated with a custom multi-well holder and fine-tuning stage for multiplexed, high-throughput SERS analysis.

The experimental workflow for detecting and distinguishing various *E. coli* serotypes using SERS is illustrated in Figure 1. Silver nanorod (AgNR) substrates were fabricated via GLAD, producing highly ordered arrays with uniform morphology and densely distributed plasmonic “hot spots.” These features maximize SERS enhancement by significantly amplifying the local electromagnetic field (|*E*|^4^) around the nanorods, thereby boosting the Raman signal of adsorbed analytes. To achieve bacterial specificity, the AgNR substrates were functionalized with VAN by immersion, forming a VAN coating on the nanorod surfaces. VAN selectively binds to the D-Ala-D-Ala termini in bacterial cell wall peptidoglycans, enhancing both selectivity and capture efficiency [26,27]. This functionalization ensures uniform binding of target cells, reduces signal variability, and minimizes non-specific adsorption, which is particularly beneficial for analyzing heterogeneous samples with multiple *E. coli* strains or varying cell concentrations. The VAN-coated AgNRs were incubated with bacterial suspensions for 2 h to enable efficient bacterial capture.

The study focused on seven clinically and environmentally significant *E. coli* serotypes—O26:H11, O157:H7, O111, O145:NM, O103:H2, O121:H7, and O45:H2. These serotypes, differentiated by their O (somatic) and H (flagellar) antigens, exhibit high genetic and physiological similarity, all belonging to the same species. Notably, serotypes such as O157:H7, O26:H11, and O145:NM are Shiga toxin-producing *E. coli* (STEC), which are important due to their association with severe illnesses like hemorrhagic colitis and hemolytic uremic syndrome. Their inclusion underscores their epidemiological relevance in foodborne outbreaks and public health surveillance. Discriminating among closely related serotypes is challenging because traditional microbiological and biochemical assays often lack sufficient resolution, given the conserved cell wall structures and overlapping metabolic profiles.

SERS spectra were collected using a portable handheld Raman spectrometer (Rapid ID system, with a weight of 919 g and a dimension of 19.6 × 11.4 × 6.1 cm^3^), emphasizing the system’s suitability for rapid, on-site analysis. For each strain and concentration, five replicate measurements were performed on two independently prepared substrates, ensuring robust statistics and reproducibility. All 10 measurements were completed in under 2 min, highlighting the method’s efficiency. As depicted in Figure 1 (lower left), the setup integrates the portable spectrometer with a custom specimen holder containing a linear array of SERS substrates (1 × 4 Ag nanorod wells). The holder is mounted on an adapter stage, enabling precise, sequential alignment of each well with the excitation laser for efficient multiplexed analysis without repeated realignment. A micro-tuning knob allows fine adjustment of the laser position within each well, enabling spectral acquisition from multiple locations and reducing substrate heterogeneity effects. The modular, scalable design supports high-throughput applications and can be adapted for automation by incorporating a motorized stage and electronic controls, further enhancing the system’s potential for reproducible, high-throughput SERS analysis in both research and applied settings. Although a 2 h incubation step is used to maximize bacterial capture on the VAN-modified surface, the subsequent SERS acquisition and classification take less than 2 min, yielding an overall analysis time (~2 h) still considerably shorter than standard culture or PCR methods [3,4,5,6,12].

### 2.2. Optimization of AgNR Substrates

**Figure 2:**
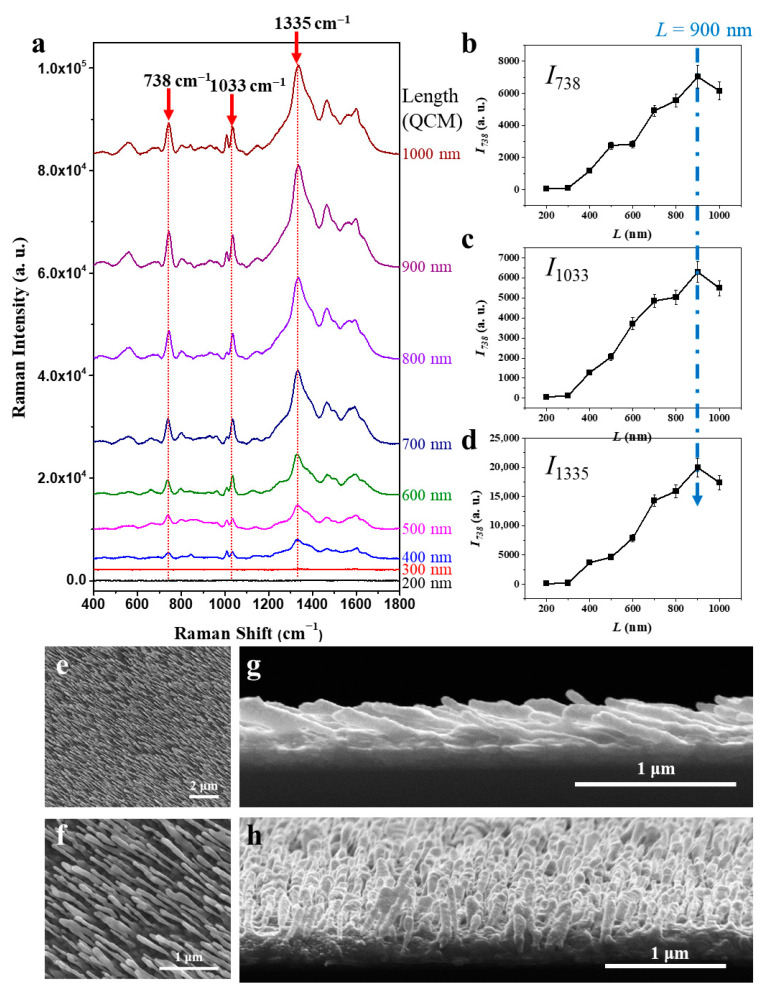
Optimization of AgNR length for SERS-based bacterial detection. (**a**) SERS spectra of *E. coli* K12 on AgNR substrates with different QCM-monitored thicknesses (200–1000 nm), highlighting key bacterial Raman peaks at ∆v= 738, 1033, and 1335 cm^−1^. (**b**–**d**) Quantitative dependence of SERS intensity at these peaks on AgNR thickness, showing maximal enhancement at t= 900 nm. (**e**–**h**) SEM images of AgNR substrates (nominal 900 nm thickness) displaying uniform, densely packed, vertically aligned nanorods with high aspect ratio.

The length of AgNRs is a critical parameter in designing SERS substrates for bacterial detection. Traditionally, longer AgNRs are used to maximize sensitivity for small molecules, as these can easily diffuse into the nanorod gaps and access plasmonic “hot spots” with enhanced electromagnetic fields (|*E*|^4^), resulting in strong SERS signal amplification. However, this approach is not optimal for larger biological targets like bacteria, which, due to their size (typically 1–2 μm long and 0.5–1 μm in diameter), cannot penetrate the nanorod interstices. Instead, bacteria mainly interact with the tips of the nanorods, reducing the effective contact area and limiting SERS enhancement. Therefore, optimizing AgNR length is essential for maximizing bacterial contact with the substrate, thereby improving SERS signal intensity and lowering the detection limit. AgNR length is directly controlled by the silver deposition thickness, monitored in real time using a quartz crystal microbalance (QCM). Systematic variation of AgNR length is necessary to determine the optimal configuration for bacterial detection. Figure 2a presents a comparative analysis of SERS spectra from *E. coli* K12 on AgNR substrates with different nanorod lengths, corresponding to QCM-monitored thicknesses of t = 200, 300, 400, 500, 600, 700, 800, 900, and 1000 nm. For each substrate, a fixed droplet (3 × 10^7^ CFU/mL) of *E. coli* K12 was applied, and SERS spectra were recorded under identical conditions to assess the impact of AgNR length on signal intensity. Across all substrates, prominent and well-established bacterial Raman peaks were consistently observed at ∆v= 738, 1033, and 1335 cm^−1^ (marked by vertical red arrows in Figure 2a). These correspond to key molecular vibrations in bacterial cells—adenine ring breathing (∆v= 738 cm^−1^, nucleic acids) [28], phenylalanine (∆v= 1033 cm^−1^, proteins) [29], and tryptophan (∆v= 1335 cm^−1^, amino acid in bacterial proteins) [30]—confirming their assignment to *E. coli*.

Figure 2b–d quantitatively show the dependence of SERS intensities at ∆v= 738, 1033, and 1335 cm^−1^ on AgNR thickness (t = 200–1000 nm, as measured by QCM). The results reveal a non-linear relationship: SERS intensity increases with nanorod length, peaking at around t = 900 nm, and then slightly decreases with further increases in thickness. This trend is attributed to the geometric mismatch between bacteria and the nanorod arrays—longer nanorods reduce the density or accessibility of plasmonic hot spots at the tips, limiting effective bacterial contact and diminishing signal enhancement. The observed SERS intensity, $I_{\mathrm{SERS}}$, can be expressed as follows:

(1) $$\;I_{\mathrm{SERS}}\;\propto N\;\times {\left|E\right|}^{4}\;\times A_{\mathrm{contact}}$$

where *N* is the number of bacteria effectively contacting the nanorod tips, ${\left|E\right|}^{4}$ is the local electromagnetic field enhancement, and $A_{\mathrm{contact}}$ is the effective contact area. For longer nanorods, $A_{\mathrm{contact}}$ does not increase proportionally, and the distribution of hot spots becomes less favorable for large targets like bacteria, resulting in reduced overall enhancement. Figure 2e–h present SEM images of AgNR substrates with t = 900 nm, providing detailed morphological characterization. Top-view SEMs (Figure 2e,f) show a uniformly distributed, densely packed, and highly ordered array. Energy dispersive spectroscopy (EDS) data confirmed that the nanorod array consists predominantly of Ag without significant impurities (Figure S1, Supporting Information (SI)). At higher magnification (Figure 2f), individual nanorods are clearly resolved, highlighting their cylindrical shape and regular spacing. Image analysis indicates an average density of ~18 rods/μm^2^. Cross-sectional SEMs (Figure 2g,h) confirm that the nanorods are vertically aligned with minimal tilt or curvature. Morphometric measurements yield average diameters and lengths of ~60 nm and ~500 nm, respectively, corresponding to an aspect ratio of ~8.3. This high aspect ratio increases surface area and promotes the formation of dense plasmonic hot spots at the nanorod tips and gaps, significantly enhancing SERS activity. Our goal was to identify an array geometry that maximizes SERS enhancement for rod-shaped *E. coli*, and the optimization with one representative strain is generally transferable to related serotypes, as SERS efficiency depends mainly on substrate morphology, surface chemistry, and cell–substrate interactions rather than minor serotype-specific differences.

### 2.3. Optimization of VAN Coating

**Figure 3:**
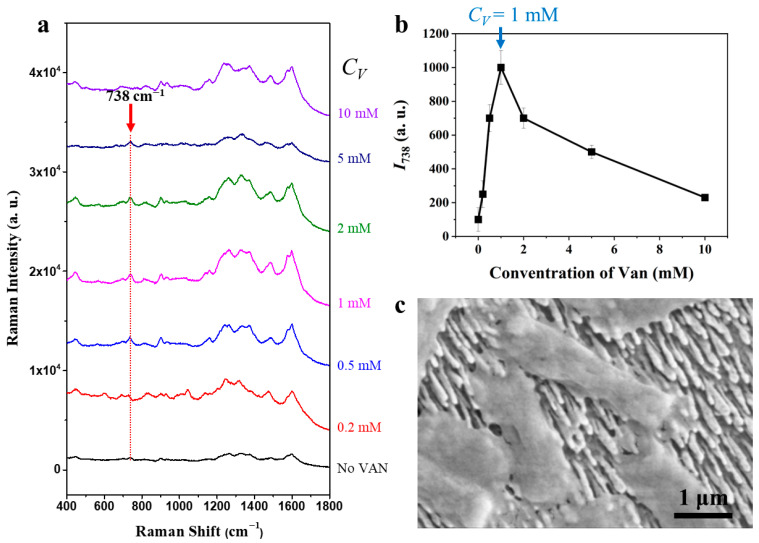
Effect of VAN concentration on bacterial capture and SERS performance. (**a**) SERS spectra of *E. coli* K12 on AgNR substrates (900 nm) coated with varying VAN concentrations (0–10 mM), showing intensity changes at the ∆v= 738 cm^−1^ peak; (**b**) quantitative plot of 738 cm^−1^ peak intensity as a function of VAN concentration, with maximal SERS signal at 1 mM; (**c**) SEM image illustrating efficient and uniform immobilization of rod-shaped *E. coli* cells on the VAN-coated AgNR substrate.

VAN was employed as an affinity ligand to enhance bacterial capture efficiency, a strategy widely applied in biosensing for specific and targeted cell recognition on functionalized surfaces [12,31,32,33,34,35]. The immobilization of VAN on Ag nanorods primarily occurs through coordination between Ag surface atoms and the oxygen/nitrogen donor atoms of VAN, forming Ag-O and Ag-N linkages [36,37,38]. These coordination bonds, together with auxiliary hydrogen bonding, ensure stable attachment. Importantly, the outward-exposed D-Ala-D-Ala binding pocket retains its specific hydrogen-bonding and steric complementarity with bacterial peptidoglycan precursors [39], thereby enabling selective and robust bacterial attachment. Immobilization of VAN on nanostructured Ag substrates thus forms an effective affinity layer that enriches target bacteria at the sensor interface, ultimately improving SERS detection sensitivity. However, the performance of VAN -functionalized surfaces is highly dependent on the density and spatial distribution of VAN molecules. Too little VAN may result in poor bacterial capture, reducing sensitivity, while excessive VAN can cause multilayer formation or overcrowding, blocking SERS-active sites and impeding effective contact between bacteria and the plasmonic substrate. Overcoating may also introduce Raman background signals or attenuate the electromagnetic enhancement essential for SERS. Thus, optimizing VAN concentration is crucial to balance maximum bacterial binding with optimal SERS signal enhancement. In this study, the impact of VAN concentration on bacterial capture and SERS signal intensity was systematically evaluated using AgNR substrates (t = 900 nm). AgNRs were coated with VAN at concentrations of 0, 0.2, 0.5, 1, 2, 5, and 10 mM, then incubated with *E. coli* K12 (10^7^ CFU/mL). After washing and drying, SERS spectra were acquired under identical conditions. As shown in Figure 3a, the SERS intensity of the characteristic bacterial Raman peak at ∆v= 738 cm^−1^ (adenine ring breathing) increases with VAN concentration, indicating improved bacterial capture. However, the VAN coating itself exhibits Raman peaks in the 1200–1600 cm^−1^ range [12], which can overlap with and obscure the bacterial peak at ∆v= 1335 cm^−1^. Additionally, the ∆v= 1033 cm^−1^ bacterial peak becomes less distinct after VAN coating, likely due to spectral overlap or masking by the VAN layer.

Figure 3b quantitatively plots the intensity *I*_738_ at ∆v= 738 cm^−1^ as a function of VAN concentration *C*_V_. The results show that SERS signal increases with VAN concentration up to 1 mM, reflecting enhanced bacterial capture due to higher ligand density and more efficient affinity-based binding. However, at concentrations above 1 mM, SERS intensity decreases. The bacterial capture efficiency of the VAN-coated AgNRs increases markedly as the VAN concentration rises (Figure S3c, Supplementary Information). This reduction is likely caused by excessively dense or multilayered VAN coatings, which can block direct contact between bacterial cells and SERS-active AgNR regions or introduce steric hindrance, thereby impeding optimal cell-surface interaction. Overcoating may also increase the distance between bacteria and the plasmonic surface, weakening the electromagnetic enhancement |*E*|^4^ necessary for strong SERS signals. Figure 3c presents a SEM image showing rod-shaped bacterial cells immobilized on the AgNR substrate. The VAN-coated AgNRs prior to bacterial treatment is shown in Figure S2 in the Supplementary Information. The bacteria are distributed across the nanorod array and are in close contact with the vertically aligned nanorods, spanning several nanorods along their length. The AgNRs appear as densely packed, high-aspect-ratio structures-oriented perpendicular to the surface, consistent with fabrication design. This close association between bacteria and AgNRs confirms effective affinity capture, most likely mediated by VAN functionalization. The tight proximity ensures that the bacterial envelope is located within the plasmonic “hot spots,” which is essential for optimal SERS response. The SEM image also demonstrates the preservation of both bacterial and nanorod integrity after capture and washing, highlighting the robustness of the VAN coating and the sample preparation protocol. In addition, microscopic imaging (Figure S3, Supporting Information (SI)) shows that VAN functionalization (1 mM) enabled efficient bacterial capture (~150 cells), in stark contrast to the unmodified surface (~3 cells). In this work, VAN was employed not for its bactericidal action, but for its high-affinity recognition of the D-Ala–D-Ala motifs, enabling selective bacterial capture on the Ag-coated arrays independent of MIC values. Instead, capture efficiency is determined by the optimized VAN coating (1 mM) and standardized incubation/washing protocols, which ensured reproducible SERS intensities across serotypes. Furthermore, the VAN coating protects the Ag surface by blocking nonspecific adsorption of contaminants or metabolic by-products [11], ensuring high reproducibility and reliability of bacterial classification [12]. Vancomycin molecules are immobilized on AgNRs stably as supported by reproducible spectra and washing stability. In addition, the LOD for *E. coli* O26:H11 is approximately 10^7^ CFU/mL (Section S3, Supplementary Information), confirming sensitive and quantitative SERS detection with robust correlation between cell load and signal. Although this LOD is higher than some previous reports using filtrate collected method [15,40], it primarily reflects the efficiency of bacterial capture on the substrate, not the intrinsic SERS detection limit. The LOD can be further improved by enhancing the substrate’s ability to enrich and capture bacteria within a given sample volume.

### 2.4. Bacterial Identification

**Figure 4:**
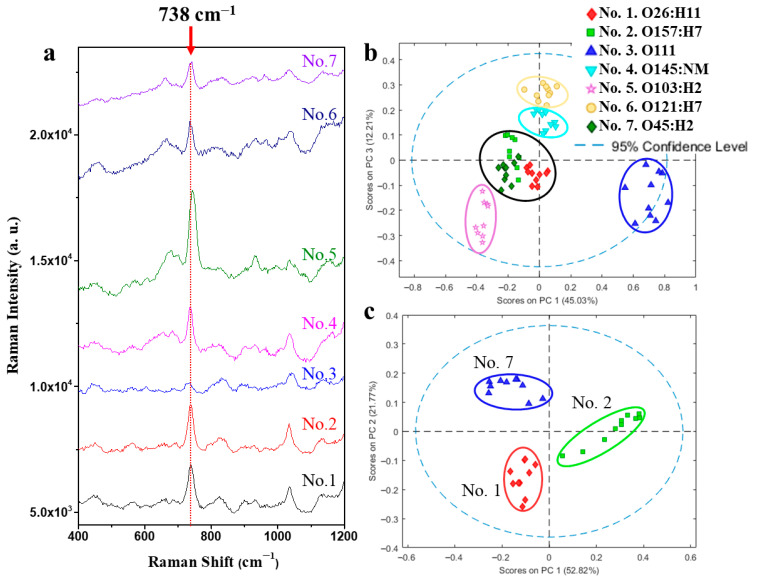
SERS-based discrimination of seven *E. coli* serotypes on optimized AgNR substrates (900 nm, 1 mM VAN). (**a**) Overlaid SERS spectra (400–1200 cm^−1^) of O26:H11, O157:H7, O111, O145:NM, O103:H2, O121:H7, and O45:H2 at 10^8^ CFU/mL. (**b**) PCA scatter plot for all seven serotypes showing distinct clustering for most strains, with 95% confidence ellipses. (**c**) Focused PCA of O26:H11, O157:H7, and O45:H2 demonstrating improved separation of these closely related serotypes.

In this study, we specifically focused on discriminating pure cultures of different *E. coli* serotypes and quantifying their absolute concentrations. The bacterial cells were thoroughly washed and resuspended in sterile water prior to SERS analysis, ensuring that the observed spectra reflect only cell-surface signatures and metabolites, without interference from culture medium components. Figure 4a presents the overlaid SERS spectra of seven distinct *E. coli* serotypes (O26:H11, O157:H7, O111, O145:NM, O103:H2, O121:H7, and O45:H2), each measured at 10^8^ CFU/mL using AgNR substrates optimized for bacterial detection (900 nm thickness, 1 mM VAN coating). Unique colors distinguish the spectra for each serotype. The reproducible and comparable levels of capture and spectral quality observed across these strains confirmed that the optimized nanorod geometry was robust and not limited to the K12 model. Control measurements of the VAN coating alone show significant peaks only in the 1200–1600 cm^−1^ region, with minimal interference in the 400–1200 cm^−1^ window (Figure S5, Supplementary Information). Therefore, analysis focuses on 400–1200 cm^−1^ to ensure spectral features primarily arise from the bacterial cells. Across all strains, a prominent peak at ∆v= ~738 cm^−1^ (adenine ring breathing) is consistently observed (red arrow). Additional differences in peak intensity and position at other wavenumbers reflect serotype-specific molecular composition, such as variations in membrane proteins, lipids, and polysaccharides, enabling discrimination between closely related *E. coli* serotypes by SERS. Figure 4b shows PCA results for the seven serotypes. The scatter plot displays distinct clustering for most strains, with each group marked by unique symbols and 95% confidence ellipses. Strains O111, O145:NM, O103:H2, and O121:H7 show clear separation, highlighting substantial inter-strain spectral variability and supporting the discriminative potential of SERS with multivariate analysis. However, the clusters for O26:H11, O157:H7, and O45:H2 overlap significantly, indicating similar SERS spectral profiles and making these strains more difficult to differentiate. To address this, a secondary PCA (Figure 4c) was performed on these three strains alone, resulting in improved cluster separation and minimal overlap between confidence ellipses, thus enhancing identification accuracy for these closely related serotypes. The loading plots of the PCA in Figure 4b,c were shown in Figure S6 in Supplementary Information. Similar limitations were observed across other tested concentrations (Figure S7, Supplementary Information), where PCA was unable to fully resolve all serotype clusters due to varying degrees of inter-group overlap. Moreover, the complex variations in SERS spectra arising from different concentrations and strains prevent PCA from simultaneously achieving accurate strain identification and concentration determination within a single model. Collectively, these results demonstrate that PCA alone is insufficient for robust and reliable classification, underscoring the need for more advanced analytical algorithms.

**Figure 5:**
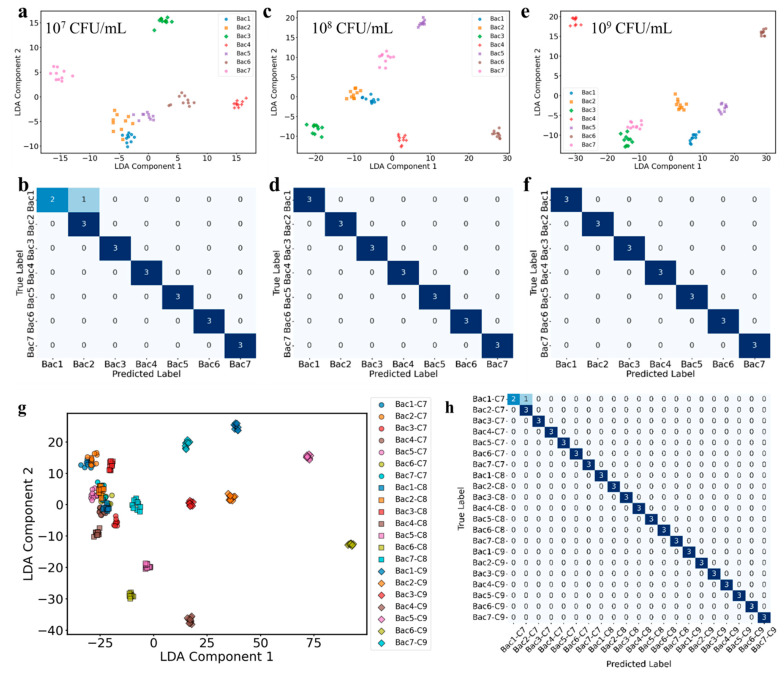
LDA scatter plots (**a**,**c**,**e**) and corresponding confusion matrices (**b**,**d**,**f**) illustrate the classification of seven *E. coli* serotypes (O26:H11, O157:H7, O111, O145:NM, O103:H2, O121:H7, and O45:H2) at individual concentrations of 10^7^, 10^8^, and 10^9^ CFU/mL. Panels (**g**,**h**) present the results from a unified LDA model constructed using all three concentrations and all serotypes together.

To improve bacterial classification, we subsequently employed linear discriminant analysis (LDA) [41], a supervised dimensionality reduction technique that maximizes the separation between predefined classes. Initial examination of the raw spectral data revealed substantial high-frequency noise, which could potentially affect subsequent feature extraction and model performance. To address this, a Savitzky–Golay (SG) filter [42] was applied to the raw data, effectively smoothing the spectra, suppressing noise, and preserving the primary spectral features (Section S6, Supplementary Information). Nevertheless, even after SG filtering, PCA still did not provide adequate discrimination among the classes (Section S7, Supplementary Information), further motivating the use of LDA for enhanced classification performance. Figure 5a–f shows the results of LDA applied to the SERS spectra of seven *E. coli* serotypes—O26:H11, O157:H7, O111, O145:NM, O103:H2, O121:H7, and O45:H2—across three bacterial concentrations: 10^7^ (a, b), 10^8^ (c, d), and 10^9^ (e, f) CFU/mL. For each concentration, the top panels (a, c, e) display LDA scatter plots of the first two canonical variables, while the bottom panels (b, d, f) provide the corresponding confusion matrices that summarize classification outcomes. Inspection of the LDA scatter plots reveals clear and well-separated clusters for all serotypes at each tested concentration. At 10^7^ CFU/mL, six out of seven serotypes form distinct groups, with only minimal overlap, as corroborated by the confusion matrix indicating a classification accuracy of 95.24%. At higher concentrations, both 10^8^ and 10^9^ CFU/mL, all serotypes are perfectly discriminated, evidenced by the complete separation of clusters in the LDA space and the corresponding confusion matrices showing 100% accuracy. These results highlight the superior discriminatory power of LDA compared to PCA, particularly when distinguishing between serotypes with highly similar SERS spectral profiles. While PCA revealed significant overlaps among O26:H11, O157:H7, and O45:H2 at certain concentrations, LDA successfully resolves these clusters, even at the lowest concentration tested. The enhanced performance of LDA can be attributed to its supervised nature, which maximizes the ratio of between-class variance to within-class variance, as expressed by the Fisher criterion [41]:

(2) $$J\left(w\right)\;=\;\frac{w^{T}\;S_{b}\;w}{w^{T}\;S_{w}\;w}$$

where *S_b_* and *S_w_* are the between-class and within-class scatter matrices, respectively, and w is the linear discriminant vector.

In addition to developing separate models for distinguishing bacteria at individual concentrations, a single LDA-based model was constructed to enable the simultaneous identification of both bacterial serotype and concentration. In this integrated approach, each serotype at each concentration was treated as a distinct class, resulting in a total of 21 classes (7 serotypes × 3 concentrations). The LDA scatter plot reveals clear separation among the majority of these 21 classes, with minimal overlap observed between most clusters (Figure 5g). This effective discrimination is further supported by the confusion matrix (test set, 7:3 training-to-testing ratio), which indicates an overall classification accuracy of 98.41% (Figure 5h). Only a small number of misclassifications were detected, demonstrating that the model maintains robust performance despite the increased complexity arising from the concurrent classification of serotype and concentration. The high accuracy achieved by this comprehensive LDA model highlights its ability to disentangle the combined effects of concentration-dependent and strain-specific spectral variations. This contrasts with the PCA approach, which, as previously discussed, struggled to simultaneously differentiate serotypes and concentrations due to its unsupervised nature and the presence of spectral overlap. Collectively, these results demonstrate that LDA, when trained on a dataset encompassing multiple concentrations and serotypes, can accurately classify both attributes simultaneously. In addition, Shiga toxin-producing *E. coli* (STEC), particularly O157:H7, can cause infection at very low doses (≈10^2^–10^3^ CFU/g in contaminated food), whereas in this proof-of-concept study we tested 10^7^–10^9^ CFU/mL and demonstrated accurate discrimination of serotypes and concentration levels (Figure 4 and Figure 5); although higher than clinical levels, these results confirm the platform’s capability, and sensitivity may be further improved through standard pre-concentration or enrichment steps commonly applied in clinical microbiology.

## 3. Conclusions

In summary, we have developed a robust and highly sensitive SERS-based platform for the rapid detection and discrimination of multiple clinically relevant *Escherichia coli* serotypes. By systematically optimizing the length of Ag nanorod arrays fabricated via glancing angle deposition and the density of VAN functionalization, we achieved efficient and selective bacterial capture along with strong and reproducible SERS enhancement. The optimized substrates, integrated with a portable Raman spectrometer and custom high-throughput sample handling, enabled direct fingerprinting of seven *E. coli* serotypes. Advanced multivariate analysis using linear discriminant analysis (LDA) allowed for accurate classification of both serotype identity and bacterial concentration. An overall classification accuracy of 98.41% was achieved across combined data from all serotypes and concentrations, with up to 100% accuracy observed in single-concentration analyses. The novelty of this work lies in integrating vancomycin-modified AgNR substrates with a supervised LDA model for rapid and quantitative SERS-based serotype identification. This work not only advances the practical utility of SERS for on-site pathogen monitoring, but also demonstrates the potential for simultaneous qualitative and quantitative bacterial analysis using a portable, user-friendly platform. Future efforts to further enhance capture efficiency and lower detection limits, as well as expand the platform to other pathogens and real-world samples, will accelerate the translation of this technology into food safety, clinical diagnostics, and environmental monitoring applications.

## 4. Experimental Section

### 4.1. Fabrication of Ag Nanorod Substrates

Ag nanorod (AgNR) array substrates were fabricated by the glancing angle deposition (GLAD) method using a custom electron beam evaporation system (Figure S10, Supplementary Information). Cleaned glass slides were first coated with a 20 nm titanium adhesion layer at 0.2 nm/s, followed by a 200 nm silver layer at 0.3 nm/s, both deposited with the substrate perpendicular to the vapor flux. For nanorod growth, the substrate was tilted to 86° relative to the vapor source, and silver was deposited at 0.3 nm/s. Film thickness was continuously monitored with a quartz crystal microbalance (QCM). To optimize bacterial detection, the AgNR layer thickness was adjusted from 200 nm to 1000 nm.

### 4.2. Bacteria Functionalization and SERS Measurement

*Escherichia coli* strains were obtained from the Center for Food Safety, The University of Georgia (Griffin, GA). Long-term preservation was carried out at −80 °C in 15–20% glycerol, and isolates were revived on trypticase soy agar (TSA) slants maintained at 4 °C for short-term storage. For experimental use, colonies from TSA slants were inoculated into trypticase soy broth (TSB, Difco) and incubated overnight at 37 °C for 18–24 h. The resulting stationary-phase cultures were centrifuged, washed three times with sterilized deionized water, and resuspended in sterilized deionized water to prepare working suspensions for SERS measurements.

To prepare VAN-functionalized AgNR substrates, the as-prepared AgNR substrates were immersed in VAN solutions and incubated overnight (>12 h) to allow sufficient surface functionalization. For bacterial capture, the VAN-functionalized substrates were incubated in 1 mL of bacterial suspension at 37 °C with shaking at 200 rpm for 2 h. After incubation, the substrates were rinsed thoroughly with sterilized water to remove non-adherent bacteria and dried under ambient conditions. SERS measurements were performed using a handheld Raman spectrometer equipped with a 785 nm excitation laser (Model HRC-10HT, Enware Optronics Inc., Irvine, CA, USA). The laser power at the sample was set to 27 mW. SERS spectra were collected over the range of ~400–2000 cm^−1^ with an integration time of 300 ms. For each bacterial isolate, five spectra were acquired from each substrate, and two independent substrates were measured per isolate to ensure reproducibility.

### 4.3. Machine Learning

A machine learning-based experimental workflow was established for bacterial classification under both single-concentration and combined concentration-species conditions. Raw spectral data from each sample were preprocessed using a Savitzky–Golay (SG) filter (window length 9, polynomial order 3, derivative order 0) to suppress high-frequency noise while retaining key spectral features. For single-concentration classification, SG-filtered spectra were visualized with principal component analysis (PCA) to assess group distributions, followed by dimensionality reduction and visualization with linear discriminant analysis (LDA). An LDA classification model was then trained and evaluated on data randomly divided into training and test sets (7:3 ratio), with performance assessed by classification accuracy and confusion matrices. For joint classification, spectral data from three concentrations (10^7^, 10^8^, and 10^9^ CFU/mL) were combined, with each serotype-concentration pair defined as a unique class (21 classes in total). SG filtering and LDA were applied for two-dimensional visualization, and the dataset was similarly split for training and testing. Model performance was evaluated by calculating classification accuracy and visualizing confusion matrices as heatmaps to facilitate intuitive assessment of classification outcomes.

## Data Availability

The datasets generated during the current study are available from the corresponding author upon written request.

## Supplementary Materials

The following supporting information can be downloaded at: https://www.mdpi.com/article/10.3390/bios15110740/s1, Figure S1. EDS of the AgNR substrate with t= 900 nm. Figure S2. Cross-sectional SEM image of the AgNR after 1mM VAN-coating. The AgNRs appear slightly smoother and less sharply, which can be attributed to the vancomycin layer adsorbed on their surfaces. Figure S3. Microscopic images of Ag nanorod substrates after bacterial incubation: (a) 1 mM vancomycin (VAN) coating, showing ~150 captured *E. coli* cells within the field of view; (b) no vancomycin coating, with only ~3 cells observed; (c) 5 mM VAN coating. Scale bar: 20 μm. Figure S4. Sensitivity of SERS detection for *E. coli* O26:H11 on optimized AgNR substrates (900 nm, 1 mM VAN). (a) SERS spectra for bacterial concentrations from 10^4^ to 10^9^ CFU/mL, highlighting the 738 cm^-1^ peak. (b–f) Corresponding optical microscopy images of AgNR substrates after incubation. (g) The mean and standard deviation of the SERS peak intensity *I*_728_ of *E. coli* O26:H11 at different concentrations (10^6^ to 10^9^ CFU/mL) and sterile DI water. Figure S5. The SERS spectrum of the 1mM Van-coated *t* = 900 nm AgNR substrates. Figure S6. Loading plots of the PCA in Figure 4b and Figure 4c, respectively. Figure S7. SERS-based discrimination of seven *E. coli* serotypes on optimized AgNR substrates (900 nm, 1 mM VAN). Overlaid SERS spectra (400–1200 cm−^1^) of O26:H11, O157:H7, O111, O145:NM, O103:H2, O121:H7, and O45:H2 at (a) 10^7^ and (b) 10^9^ CFU/mL. PCA scatter plot for all seven serotypes at (c) 10^7^ and (d) 10^9^ CFU/mL, with 95% confidence ellipses. Figure S8. Comparison of raw (a) and Savitzky-Golay filtered (b) spectral profiles for the first sample of each of the seven bacterial species at 10^7^ CFU/mL. Figure S9. PCA visualization of filtered spectral data for seven bacterial species at concentrations of (a) 10^7^, (b) 10^8^, and (c) 10^9^ CFU/mL. Figure S10. Schematic of the custom GLAD system used for AgNR deposition.
