# Supplementary material for: Machine Learning-Assisted SERS Platform for Rapid and Quantitative Discrimination of Shiga Toxin-Producing E. coli Serotypes
Source: Biosensors (Basel). 2025 Nov 4;15(11):740. doi: 10.3390/bios15110740 (PMC12650715; doi:10.3390/bios15110740)
Supplement: Supplementary file 1 [file biosensors-15-00740-s001.zip › biosensors-3907889-supplementary.pdf]

Machine Learning-Assisted SERS Platform for Rapid and Quantitative Discrimination of Shiga Toxin-Producing *E. coli* Serotypes

Yuting Liu[1], *Jiyu Feng*[1], *Xinyi Chen*[1], *Mingyu Cheng*[1], *Jinglan Zhang*[1], *Xu Ye*[1], Yiping Zhao[2], and *Bin Ai*[1*]

[1] Chongqing Key Laboratory of Bio-Perception & Intelligent Information Processing, School of Microelectronics and Communication Engineering, Chongqing University, Chongqing 400044, China

[2] Department of Physics and Astronomy, The University of Georgia, Athens, GA 30602, USA

* Correspondence: binai@cqu.edu.cn

Section S1. EDS of the AgNR

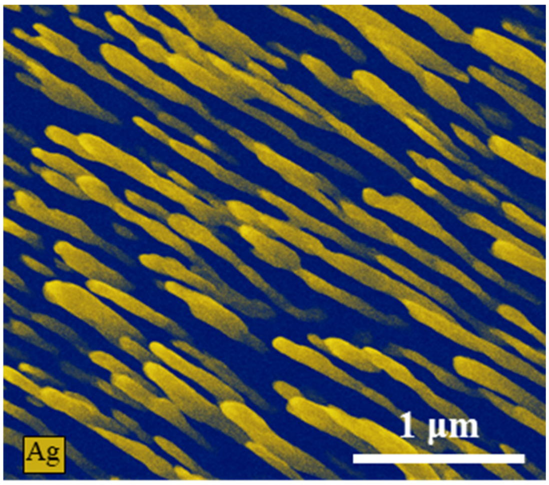

Figure S1. EDS of the AgNR substrate with $t = 900$ nm.

Section S2. Images of cell adhesion

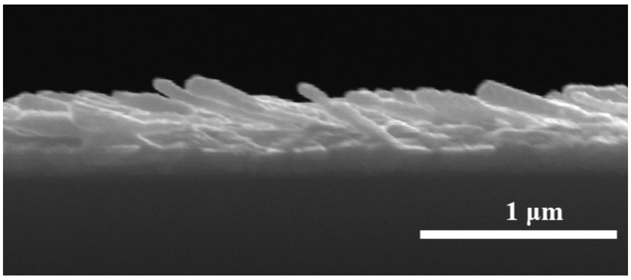

Figure S2. Cross-sectional SEM image of the AgNR after 1mM VAN-coating. The AgNRs appear slightly smoother and less sharply, which can be attributed to the vancomycin layer adsorbed on their surfaces.

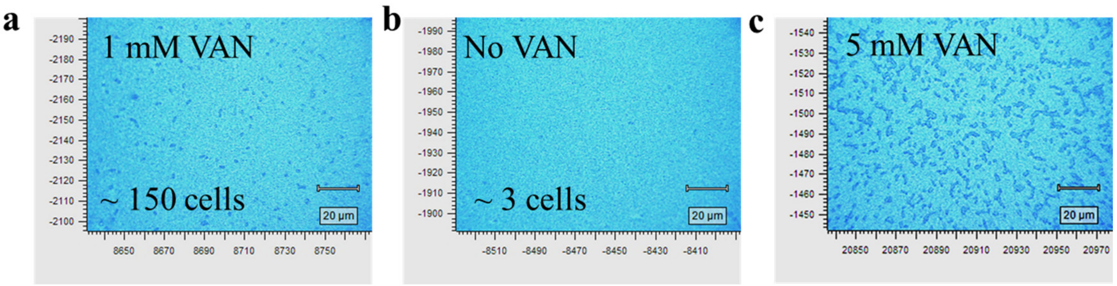

Figure S3. Microscopic images of Ag nanorod substrates after bacterial incubation: (a) 1 mM vancomycin (VAN) coating, showing ~150 captured *E. coli* cells within the field of view; (b) no vancomycin coating, with only ~3 cells observed; (c) 5 mM VAN coating. Scale bar: 20 μm.

Section S3. Limit of Detection

Figure S4a presents the SERS spectra of E. coli O26:H11 acquired using AgNR substrates optimized for bacterial detection (900 nm thickness, 1 mM VAN coating, as previously determined). SERS measurements were performed on samples with $^{-1}$ O26:H11 concentrations ranging from 10^4 to 10^9 CFU/mL, alongside a sterile DI water control. The control spectrum shows no discernible Raman peaks in the 400–1200 cm^{-1} region, indicating minimal spectral interference from the VAN coating. In contrast, samples with increasing bacterial concentrations exhibit progressively more intense and defined Raman features. Notably, the characteristic bacterial peak at 738 cm^{-1} (red arrow) shows a clear positive correlation with concentration above 10^7 CFU/mL; at concentrations of 10^6 CFU/mL or lower, this peak is not detected. **Figures S4b–f** display optical microscopy images of VAN - functionalized AgNR substrates after incubation with *E. coli* O26:H11 at different initial concentrations (10^9 to 10^5 CFU/mL). The cell count per field of view decreases systematically with lower bacterial concentrations: about 400, 150, 40, and 3 cells for 10^9, 10^8, 10^7, and 10^6 CFU/mL, respectively, and no cells observed at 10^5 CFU/mL. These

microscopy findings closely match the SERS results, with the SERS peak at 738 cm^{-1} diminishing and disappearing in parallel with the reduction in captured bacteria. This strong correlation underscores the quantitative relationship between surface-bound bacterial cells and SERS signal intensity.

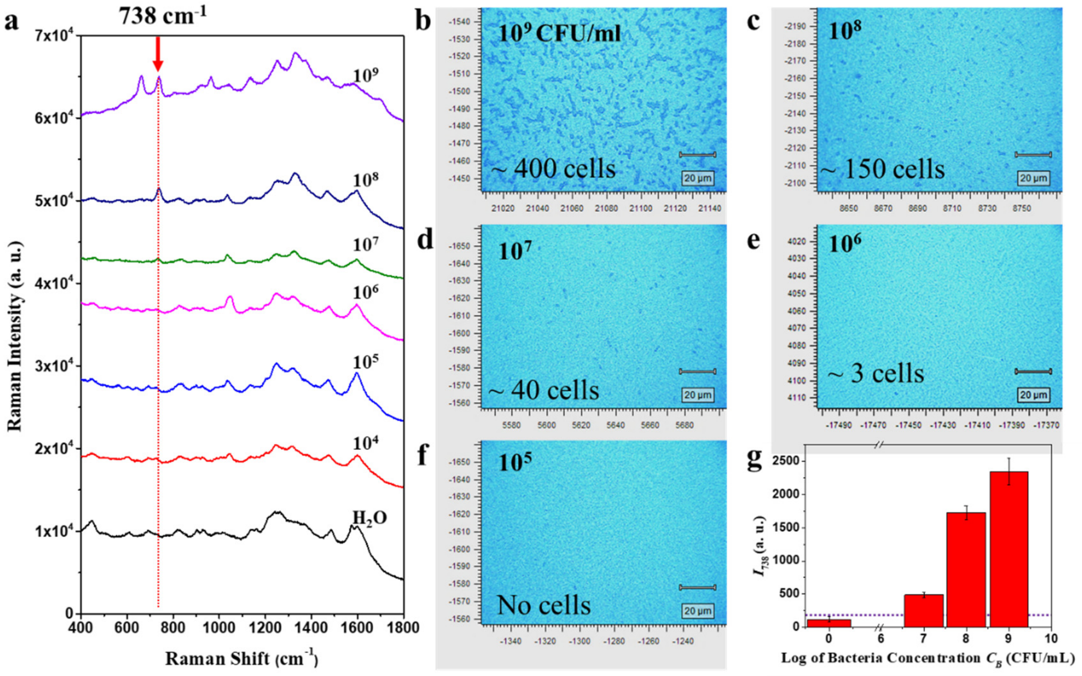

Figure S4. Sensitivity of SERS detection for *E. coli* O26:H11 on optimized AgNR substrates (900 nm, 1 mM VAN). (a) SERS spectra for bacterial concentrations from 10^4 to 10^9 CFU/mL, highlighting the 738 cm^{-1} peak. (b–f) Corresponding optical microscopy images of AgNR substrates after incubation. (g) The mean and standard deviation of the SERS peak intensity I_{728} of E. coli O26:H11 at different concentrations (10^6 to 10^9 CFU/mL) and sterile DI water.

Figure S4g quantitatively illustrates the relationship between *E. coli* O26:H11 concentration and SERS signal intensity on VAN-functionalized Ag nanorod substrates. The plot shows the mean SERS peak intensity at $\Delta\nu = 728$ cm^{-1} (I_{728}) versus the logarithm of bacterial concentration (C_B, CFU/mL), with error bars representing standard deviations. The data reveal a clear positive correlation: at low concentrations ($\leq 10^6$ CFU/mL), I_{728} is similar

to the DI water control, indicating negligible bacterial detection. As the concentration increases from 10^7 to 10^9 CFU/mL, I_{728} rises sharply and significantly, demonstrating the concentration-dependent sensitivity of the platform. The limit of detection (LOD) was established using the control intensity (120 ± 40 AU), defining the detection threshold as the mean plus three standard deviations (240 AU). Only samples with I_{728} above this threshold are deemed reliably detectable. As shown, all samples at $\geq 10^7$ CFU/mL exceed this threshold, while lower concentrations do not. Thus, the LOD for *E. coli* O26:H11 is approximately 10^7 CFU/mL, confirming sensitive and quantitative SERS detection with robust correlation between cell load and signal.

Section S4. **SERS of the Bare Van-coated AgNR Substrates**

Figure S2 shows the SERS spectrum of the vancomycin-coated Ag nanorod (AgNR) substrate (1 mM VAN, *t* = 900 nm) in deionized water, which served as the blank medium in our experiments. The recorded spectrum confirms that neither the functionalized substrate nor the aqueous medium contributes interfering Raman features, ensuring that the spectral peaks observed in subsequent measurements originate exclusively from bacterial cells.

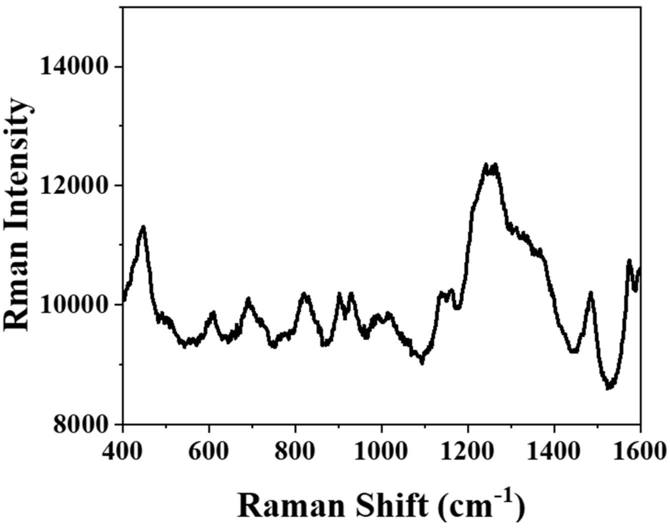

Figure S5. The SERS spectrum of the 1mM Van-coated t = 900 nm AgNR substrates.

Section S5. Bacterial Identification

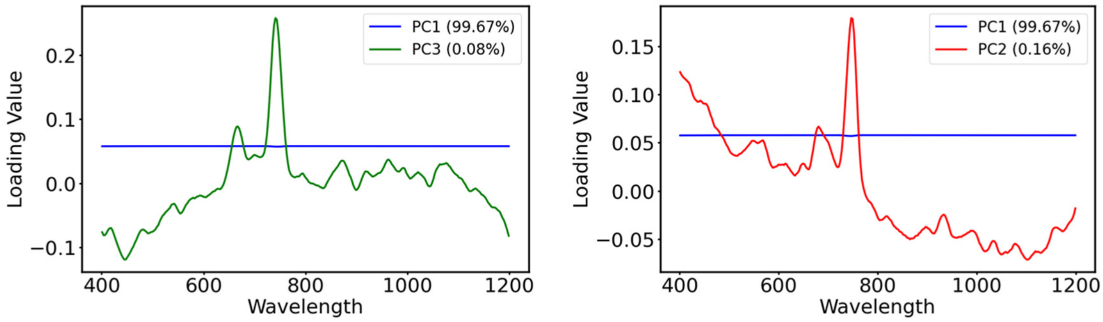

Figure S6. Loading plots of the PCA in Figure 4b and 4c, respectively.

Figure S3 summarizes the SERS analysis of seven *E. coli* serotypes (O26:H11, O157:H7, O111, O145:NM, O103:H2, O121:H7, O45:H2) using optimized Ag nanorod substrates functionalized with vancomycin. Overlaid SERS spectra at 10^7 and 10^9 CFU/mL (**Figures S3a**, **S3b**) in the 400–1200 cm^{-1} range illustrate both inter- and intra-serotype variation. PCA of these spectra (**Figures S3c**, **S3d**) shows the distribution of serotypes in the PC1–PC2 space, with 95% confidence ellipses indicating group clustering. Although some serotype separation is evident—particularly at 10^9 CFU/mL—substantial overlap remains at both concentrations, limiting robust discrimination. These results indicate that, under current conditions, PCA of SERS spectra alone is insufficient for reliable serotype classification, especially at lower concentrations, and underscore the need for more advanced analytical approaches to improve accuracy.

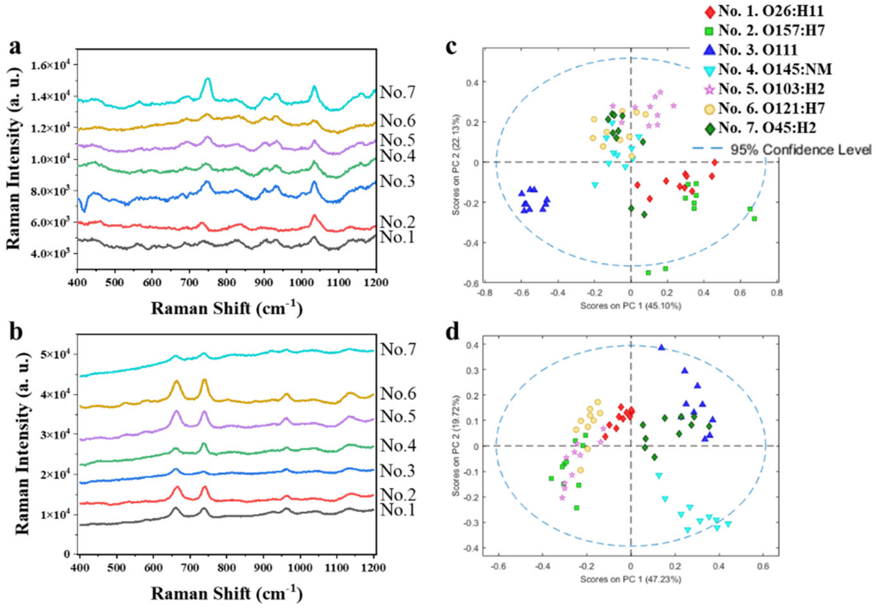

Figure S7. SERS-based discrimination of seven E. coli serotypes on optimized AgNR substrates (900 nm, 1 mM VAN). Overlaid SERS spectra (400–1200 cm^{-1}) of O26:H11, O157:H7, O111, O145:NM, O103:H2, O121:H7, and O45:H2 at (a) 10^7 and (b) 10^9 CFU/mL. PCA scatter plot for all seven serotypes at (c) 10^7 and (d) 10^9 CFU/mL, with 95% confidence ellipses.

Section S6. Savitzky-Golay (SG) Filter

The comparison of raw and Savitzky-Golay (SG) filtered spectral data for the seven bacterial species at a concentration of 10^7 CFU/mL, using the first sample of each species, reveals the effectiveness of the SG filter in noise suppression and signal preservation. In the unprocessed spectra (**Figure S4a**), substantial high-frequency noise is evident across all species, manifested as rapid, irregular fluctuations superimposed on the primary spectral features. Such noise can obscure subtle but diagnostically relevant variations between bacterial species, potentially impairing downstream classification performance. Upon application of the SG filter (**Figure S4b**), the spectral profiles exhibit marked smoothing, with noise components in the higher frequency domain significantly attenuated. The main peaks and troughs, corresponding to characteristic absorption or scattering features of each

bacterial species, are retained with minimal distortion. This balance between noise reduction and feature preservation is a key advantage of the SG filter, which employs a local polynomial regression within a moving window (window length 9, polynomial order 3 in this study) to approximate the underlying signal ($S(\lambda)$) at each wavelength (λ):

$$S_{\text{filtered}}(\lambda) = \sum_{k=-m}^{m} c_k \cdot I(\lambda + k)$$

where $I(\lambda)$ is the raw intensity at wavelength λ, c_k are the Savitzky-Golay coefficients, and m is determined by the window size.

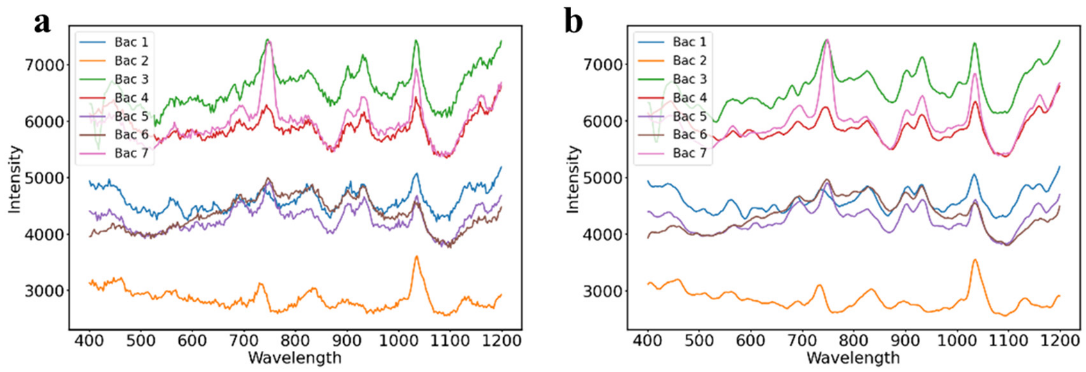

Figure S8. Comparison of raw (a) and Savitzky-Golay filtered (b) spectral profiles for the first sample of each of the seven bacterial species at 10^7 CFU/mL.

Notably, the filtered spectra enable clearer visualization and discrimination of interspecies spectral differences, particularly in regions around 400–600 nm and 1000–1200 nm, where certain bacteria display unique absorption signatures. The reduction in high-frequency noise not only enhances visual interpretability but is also expected to improve the performance of subsequent feature extraction and classification algorithms, as the signal-to-noise ratio (SNR) is substantially increased.

Section S7. PCA After SG filter

Principal component analysis (PCA) was employed to visualize the distribution of the seven bacterial species in the reduced spectral feature space after Savitzky-Golay filtering,

across three concentrations: 10^7, 10^8, and 10^9 CFU/mL, as shown in **Figure S5**. In each case, the first two principal components (PC1 and PC2) are plotted, capturing the largest variance directions in the dataset. The resulting scatter plots indicate substantial overlap among data points corresponding to different bacterial species at all tested concentrations. Specifically, at 10^7 CFU/mL (**Figure S5a**), the clusters for each species are not distinctly separated, with many instances of intermingling in the PC1–PC2 plane. Similar patterns of overlap persist as the concentration increases to 10^8 (**Figure S5b**) and 10^9 CFU/mL (**Figure S5c**). This phenomenon suggests that the variance captured by the leading principal components does not sufficiently reflect the interspecies variability, and thus, PCA alone is inadequate for effective discrimination of the bacterial groups in this spectral context.

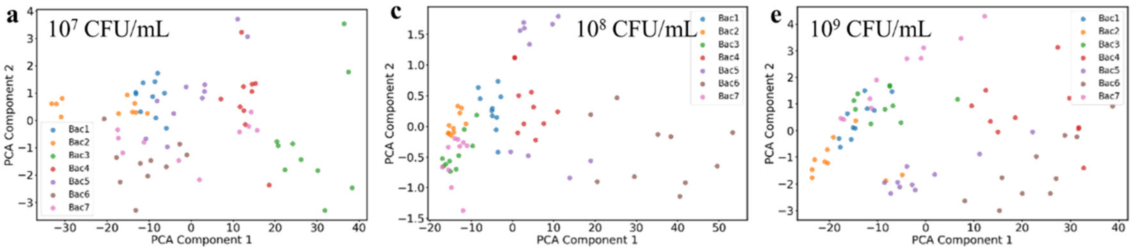

Figure S9. PCA visualization of filtered spectral data for seven bacterial species at concentrations of (a) 10^7, (b) 10^8, and (c) 10^9 CFU/mL.

The observed overlap may be attributed to the PCA algorithm’s unsupervised nature, as it seeks directions of maximal total variance rather than maximizing the separation between predefined classes. Although PCA is effective for noise reduction and data compression, its inability to exploit class label information limits its performance in this classification task. These findings underscore the necessity of employing supervised dimensionality reduction or classification techniques, such as linear discriminant analysis (LDA), which are specifically designed to enhance class separability in the feature space.

Section S8. GLAD System

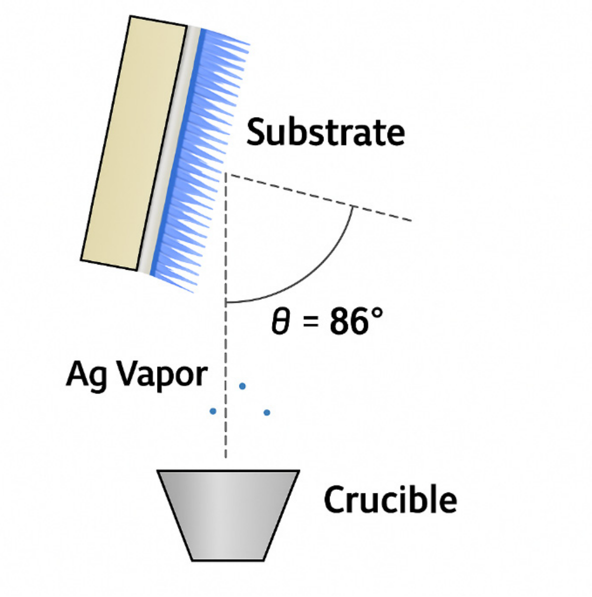

Figure S10. Schematic of the custom GLAD system used for AgNR deposition.
